# Distinct soil bacterial patterns along narrow and broad elevational gradients in the grassland of Mt. Tianshan, China

**DOI:** 10.1038/s41598-021-03937-x

**Published:** 2022-01-07

**Authors:** Rui Li, Yunhua Liu, Junhui Cheng, Nana Xue, Zongjiu Sun, Pan Zhang, Ning Li, Xiaoshuang Di, Weihua Fan, Jiang Deng, Yucheng Ma, Minfei Li, Jiandong Sheng

**Affiliations:** 1 College of Grassland and Environmental Sciences, Xinjiang Agricultural University, 311 Nongda East Road, Urumqi, 830052 Xinjiang China; 2 Key Laboratory of Soil and Plant Ecological Processes of Xinjiang, Urumqi, 830052 Xinjiang China

**Keywords:** Bacterial structural biology, Microbial ecology

## Abstract

Bacteria are essential regulators of soil biogeochemical cycles. While several studies of bacterial elevational patterns have been performed in recent years, the drivers of these patterns remain incompletely understood. To clarify bacterial distribution patterns and diversity across narrow- and broad-scale elevational gradients, we collected soil samples from 22 sites in the grasslands of Mt. Tianshan in China along three elevational transects and the overall elevation transect: (1) 6 sites at elevations of 1047–1587 m, (2) 8 sites at 876–3070 m, and (3) 8 sites at 1602–2110 m. The bacterial community diversity across the overall elevation transects exhibited a hump-like pattern, whereas consistent patterns were not observed in the separate elevational transects. The bacterial community composition at the phylum level differed across the transects and elevation sites. The Actinobacteria was the most abundant phylum overall (41.76%) but showed clear variations in the different transects. Furthermore, heatmap analyses revealed that both pH and mean annual temperature (MAT) were significantly (*P* < 0.05) correlated with bacterial community composition as well as the dominant bacterial phyla, classes, and genera. These findings provide an inclusive view of bacterial community structures in relation to the environmental factors of the different elevational patterns.

## Introduction

Elevational gradients have been studied to evaluate the relationships between altitude, spatial distribution patterns, and basic biome ecological processes in montane ecosystems^1–3^. Understanding how soil bacterial communities respond to particular environmental variables is of great ecological importance, and our current understanding of soil bacterial community elevational patterns in mountain grasslands remains relatively limited. There is thus a clear need for further research regarding bacterial elevational diversity and community composition patterns in mountain grassland ecosystems.

To date, several studies have described the patterns and drivers of soil bacterial community structures across elevational gradients in different montane regions^4–7^. For example, some studies have reported a hump-shaped pattern when evaluating soil bacterial community diversity as a function of elevation, as in a study conducted on Mount Fuji, evaluating soil bacterial diversity between 1000 and 3700 m, with a significant “peak” in bacterial diversity being detected at an elevation of approximately 2500 m^8^. Similarly found that microbes followed Humboldt diversity patterns between the elevations of 194 m and 3644 m^6^. However, some reports detected no clear patterns in soil bacterial community diversity along elevation gradients^5,9^, although decreases in soil Acidobacterial diversity have been observed as a function of elevation between 2460 and 3380 m^4^. Together, these findings suggest that bacterial composition and diversity respond differently to elevational gradients and environmental types, with bacterial communities, in particular, likely to exhibit a range of tolerance levels across different environmental conditions.

Meanwhile, bacterial community composition and diversity are known to vary substantially across montane spatial scales, and this variation is thought to be associated with changes in particular biotic or abiotic factors. The specific impacts of climatic, plant, and soil properties on bacterial community composition and diversity have been discussed in a range of studies. One widely accepted determinant of soil bacterial community distribution along elevation gradients in a range of mountain ecosystems is the soil pH^9–12^, although other studies have also suggested that other driver factors such as temperature may also be major regulators of bacterial community composition^6,13,14^. For example, studies conducted on Changbai Mountain^9^ and Segrila Mountain^12^ in China found that soil pH had a major influence on bacterial elevational patterns, whereas a separate study of an Andean transect traversing a 3.5 km elevation range determined temperature to be the predominant driver of bacterial distribution patterns^6^. Therefore, soil bacterial patterns may thus be dependent on the study scale, and the environmental stress gradient hypothesis outlines the key response strategies of soil bacteria when faced with environmental change.

Mt. Tianshan is one of the seven major mountain systems in the world, covering a large region of the Eurasian hinterland. Roughly two-thirds of the mountains in this range are located in the Xinjiang Uygur Autonomous Region of China. Besides, Mt Tianshan grassland is characterized by a typical arid and semiarid continental climate, as well as having a clear vertical zonation from desert to alpine meadow biomes. However, there is little knowledge of the shaping of bacterial communities across elevational gradients in these special habitats. In addition, several questions have emerged concerning in the patterns, drivers, and biogeographical differences of soil communities in Mt Tianshan grassland. Are there any differences in the soil bacterial communities in different areas of Mt Tianshan grassland, for instance, in different administrative regions? Due to the known impact of environmental factors in shaping of soil bacterial communities across montane spatial scales, does this relationship also exist in Mt Tianshan grassland? and, if so, what is the key factor?

To answer these questions, three typical administrative regions (transects 1, 2, and 3) were selected to study (1) the distribution patterns of soil bacteria in mountain grassland ecosystems; (2) the biogeographical differences in soil communities in the Mt Tianshan grassland; (3) the influence of environmental disturbance in shaping bacterial community diversity along the elevation gradients.

## Results

### Environmental variable quantification along an altitudinal gradient

This study area included 22 sampling sites, and 66 samples, classified into three transects, namely Transect 1 (1047–1587 m), Transect 2 (876–3070 m), and Transect 3 (1602–2110 m). Significant differences in soil properties and plant parameters were observed along the three studied altitudinal transects (*P* < 0.05) (Table 1). The soil pH ranged from 7.11 to 8.45, 5.54 to 7.82, and 5.02 to 8.83 in Transects 1, 2, and 3, respectively. In contrast to altitudinal Transects 1 and 3, the broad-scale altitudinal gradients of Transect 2(876–3070 m) exhibited more substantial changes in soil moisture (SM), soil organic carbon (SOC), total nitrogen (TN), C:N ratio (C/N), plant species richness (PSR), and belowground biomass (BGB) between sampling sites. Compared to sites situated at 876 m, 920 m, and 3070 m, the intermediate altitude site samples at 1744 m and 2513 m tended towards increased SM, TN, and SOC. In contrast, PSR and BGB were the highest in the high-altitude alpine meadow environments at 2903 m and 2981 m.

**Table 1:** Soil properties and plant variables at different sampled elevations.

Elevations (m)	pH	Soil physicochemical parameters				Plant parameters	
		SM (%)	SOC (g kg^−1^)	TN (g kg^−1^)	C:N ratio	PSR (per m^−2^)	BGB (g m^−2^)
1047	8.45a	1.20d	12.83d	1.02d	23.80a	7.67c	1106bc
1071	7.82b	2.12d	30.27c	2.83c	10.48b	11.00b	1086c
1277	7.11c	3.57c	43.15b	4.11b	10.89b	9.00bc	1011c
1423	8.29a	3.29c	12.83d	1.49d	12.14b	8.00c	2165b
1580	7.85b	10.46b	59.64a	4.69b	12.83b	15.00a	3103a
1587	7.65b	12.73a	63.97a	6.19a	10.74b	15.33a	2357a
**Transect 1**	**7.86**	**5.56**	**37.12**	**3.39**	**13.48**	**11.00**	**1805**
876	8.83a	1.21b	5.81e	0.99c	20.57a	2.67c	627e
920	8.44ab	1.63b	9.51e	1.20c	19.94a	5.67d	743de
1586	7.79b	4.04b	25.80de	2.73bc	11.45c	4.33cd	1192cd
1744	6.55c	20.29	85.92ab	6.37a	16.23b	8.67b	3099a
2513	6.14cd	17.16	65.47bc	6.47a	10.48c	9.00b	1373bc
2903	5.78cde	23.51	100.66a	6.51a	18.97a	12.00a	3427a
2981	5.02e	25.88	47.70cd	4.34ab	10.72c	13.67a	3466a
3070	5.29de	15.46	43.92cd	2.87bc	15.29b	9.00b	1725b
**Transect 2**	**6.73**	**13.65**	**48.10**	**3.94**	**15.46**	**8.13**	**1957**
1602	7.82a	9.19d	37.87c	3.79d	10.83d	14.67bc	2221bc
1661	7.64ab	19.66c	38.10c	2.83d	19.77a	7.33e	1374de
1703	6.90b	19.53c	110.38ab	10.63a	10.57d	14.33cd	1467de
1739	6.94b	19.46c	92.47b	9.07ab	10.70d	11.33d	1663cd
1998	7.40ab	8.40d	48.84c	4.24d	14.45c	11.67cd	862e
2045	5.94c	35.14a	113.00ab	10.53a	10.85d	24.00a	3029a
2075	5.99c	26.54b	94.29b	6.18c	15.43bc	22.33a	2020cd
2110	5.54c	30.07b	123.74a	7.44bc	16.88b	19.33b	2827ab
**Transect 3**	**6.77**	**21.00**	**82.34**	**6.84**	**13.69**	**15.62**	**1933**

*SM* soil moisture, *SOC* soil organic carbon, *TN* total nitrogen, *PSR* plant species richness, *BGB* belowground biomass. Values in bold indicate the mean value in the indicated Transects.

### Bacterial community composition across the altitudinal gradients

**Figure 1:**
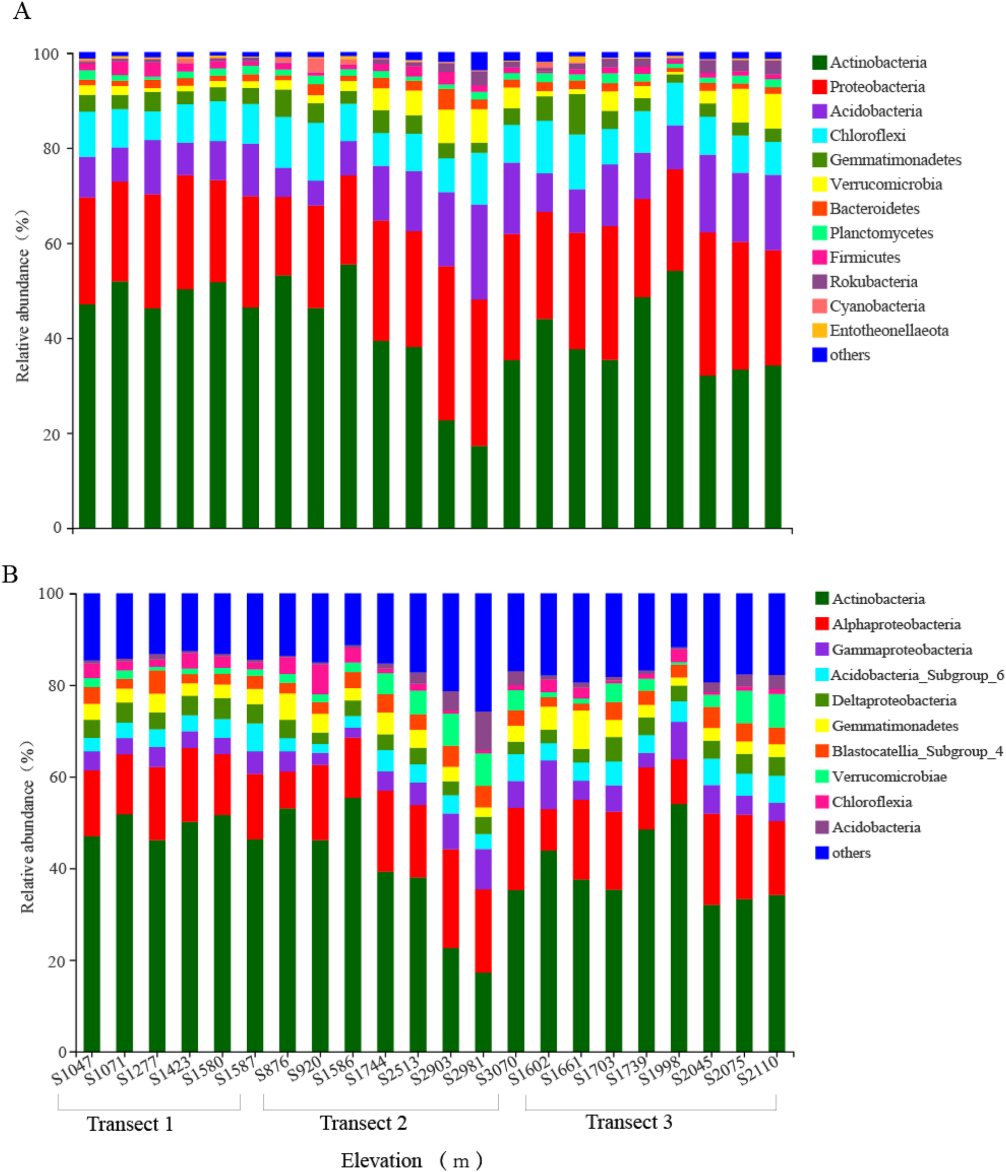
Bacterial community composition variations at the phylum (**A**) and class (**B**) levels in soil samples collected at different levels. These were done in R (v3.3.1, http://www.R-project.org).

Over the study area, the *Actinobacteria* constituted the most abundant phylum in all 22 sampling sites on Mt. Tianshan, accounting for 41.76% of total sequences, followed by the *Proteobacteria* (24.13%), *Acidobacteria* (10.85%), *Chloroflexi* (8.73%), *Gemmatimonadetes* (3.65%), *Verrucomicrobia* (3.23%), *Bacteroidetes* (1.64%) and *Planctomycetes* (1.53%) (Fig. 1A). Although there were consistent trends in soil bacterial phylum composition across samples the average relative abundance of phyla varied across elevations. For example, in Transect 2 (876–3070 m), the relative *Actinobacteria* abundance was lower in the high elevation sites (2903 m and 2981 m) relative to both the low (876 m and 920 m) and the intermediate elevation sites (1744 m and 2513 m). At the class level, a total of 11 classes were detected, with 10 having a relative abundance > 0.05%, while the remaining bacteria were merged into an “others” class. As shown in Fig. 1B, the proportion of *Actinobacteria*, *Alphaproteobacteria* and *Gammaproteobacteria* at each elevation was 45%, whereas *Deltaproteabacteria*, *Acidobacteria_Subgroup_6*, and *Gemmatimonadetes* were prevalent at low levels in most soil samples. At the genus level (Supplementary Fig. 1), 76 genera were detected in the research areas, with the dominant genera including *norank_f_*67-14_ *o_Solirubrobacterales* (5.72%), *Rubrobacter* (4.35%), *Solirubrobacter* (2.83%), *Pseudonocardia* (2.26%) and *Bradyrhizobium* (2.19%) and less than 0.01% of the bacterial genera were classified into others.

### Bacterial community composition varies along elevation gradients

**Figure 2:**
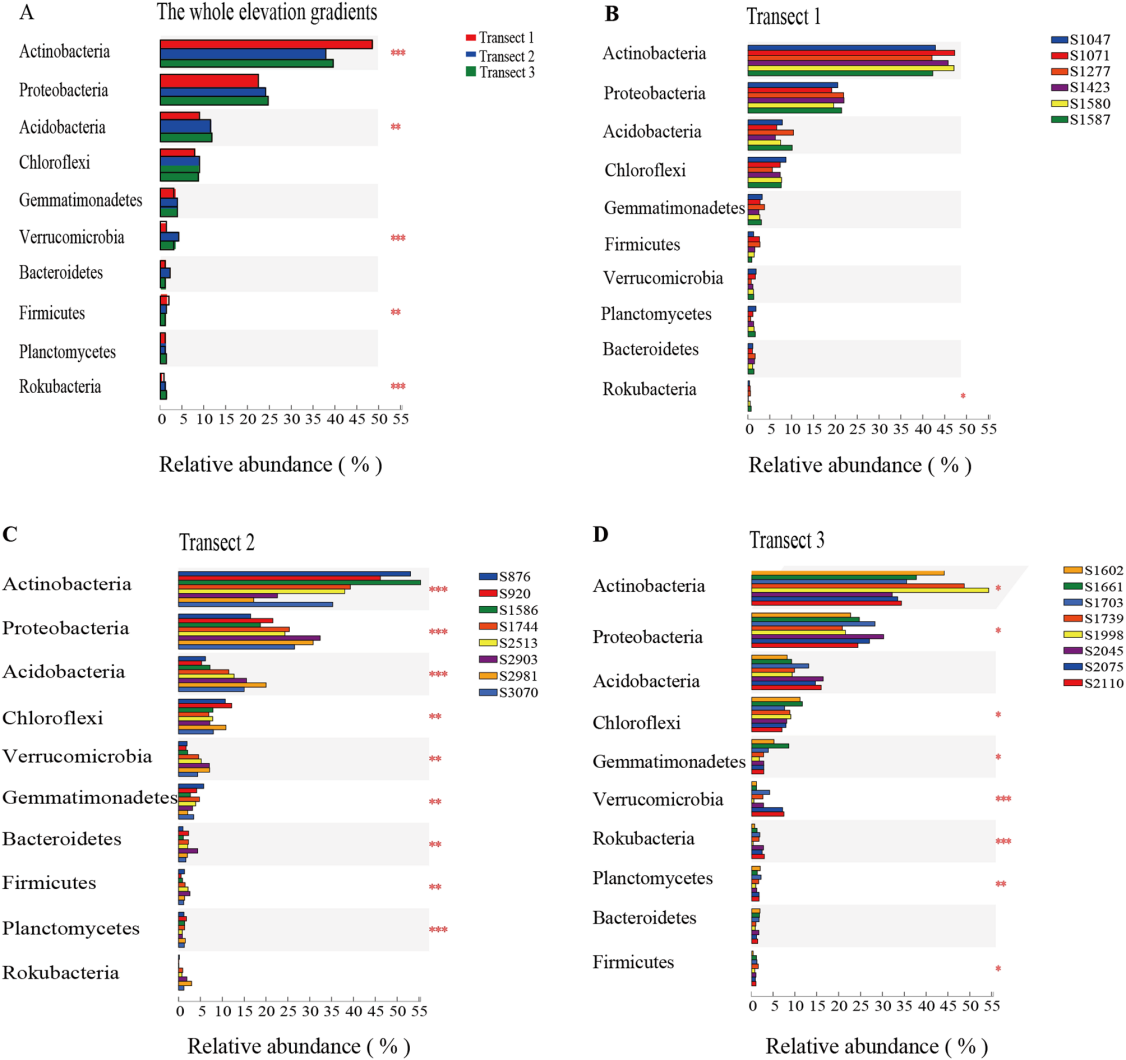
Bacterial community composition variations at the phylum level in different Transects and elevation sites. The vertical axis denotes phylum name, while the horizontal axis represents the average relative abundance in samples from different elevations, and differently colored columns represent different elevation sites. *P* values (one-way ANOVA) are marked on the right side of the graph, *P < 0.05, **P < 0.01, ***P < 0.001. These were done in R (v3.3.1, http://www.R-project.org).

We next sought to analyze the differences in relative bacterial abundance at the phylum level among Transects 1–3 (Fig. 2). Significant differences in the relative abundance of *Actinobacteria*, *Proteobacteria*, *Acidobacteria*, *Verrucomicrobia*, *Firmicutes*, and *Rokubacteria* were detected in samples from the different transects (Fig. 2A). The relative abundance of *Actinobacteria* and *Firmicutes* in Transect 1 (48.64% and 1.89%, respectively) was significantly higher than in Transect 2 (38.43% and 1.49%, respectively) and Transect 3 (39.63% and 0.98%, respectively) (*P* < 0.001 for all). In contrast, relative abundance of *Acidobacteria* and *Verrucomicrobia* in Transect 1 was lower (8.54% and 1.51%, respectively) than in Transect 2 (11.48% and 4.38%, respectively) and Transect 3 (12.06% and 3.37%, respectively) (*P* < 0.001 for all).

The relative abundance of the dominant phyla in the bacterial communities did not differ significantly among the six elevation sites (S1047, S1071, S1277, S1423, S1580, and S1587) in Transect 1, whereas significant changes were observed in the Transect 2 and Transect 3 elevation sites. The relative *Proteobacteria*, *Acidobacteria* and *Verrucomicrobia* abundance at the high elevation sites in Transect 2 and 3 (i.e., S2903 and S2981 in Transect 2, S2075 and S2110 in Transect 3) were significantly higher than those at low elevation sites in both transects (S876 and S920 in Transect 2 and S1602 and S1661 in Transect 3), whereas the opposite trend was observed for *Actinobacteria* and *Gemmatimonadetes*.

### Bacterial alpha diversity elevation patterns

**Figure 3:**
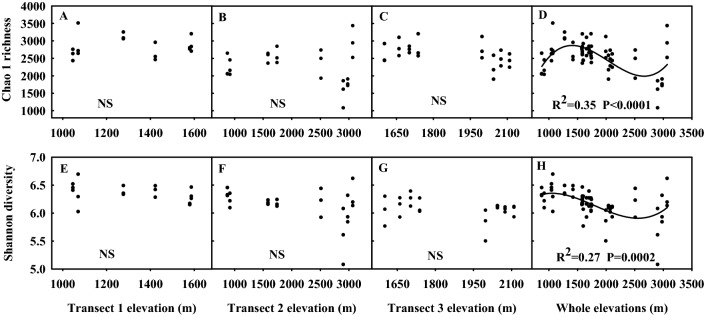
Relationships between bacteria alpha diversity and Transect 1 elevation gradients, Transect 2 elevation gradients, Transect 3 elevation gradients, and overall elevation gradients. Cubic models were tested to describe these relationships and model selection was conducted out based on R^2^ values and RMSE (root mean square error). *P* values are given to indicate significance levels. *NS* not significant. Cubic models were used SigmaPlot v 10.0 (Systat Software, San Jose, CA).

A total of 3,335,681 quality sequences across all 66 soil samples were grouped into 5314 operational taxonomic units (OTUs) at a 97% similarity level. We identified significant relationships between soil bacterial richness, diversity, and elevation (Fig. 3). No dramatic changes in the elevational bacterial diversity patterns were observed between Transects 1, 2 and 3 (Fig. 3A–C,E–G). However, across the overall elevational gradient, the Chao1 and Shannon indices exhibited pronounced hump–shaped patterns (Fig. 3D,H).

The “peak” value in Chao1 richness was detected at lower elevations at around 1423 m, while a “hollow” pattern was evident at an intermediate elevation of 2513 m (Fig. 3D). Shannon diversity decreased from 876 to 2513 m (Fig. 3H), but increased from 2513 to 3070 m. These data suggest that soil bacterial community diversity conformed to a hump-shaped profile in the Mt. Tianshan grasslands.

### Bacterial community beta diversity along elevational gradients

**Figure 4:**
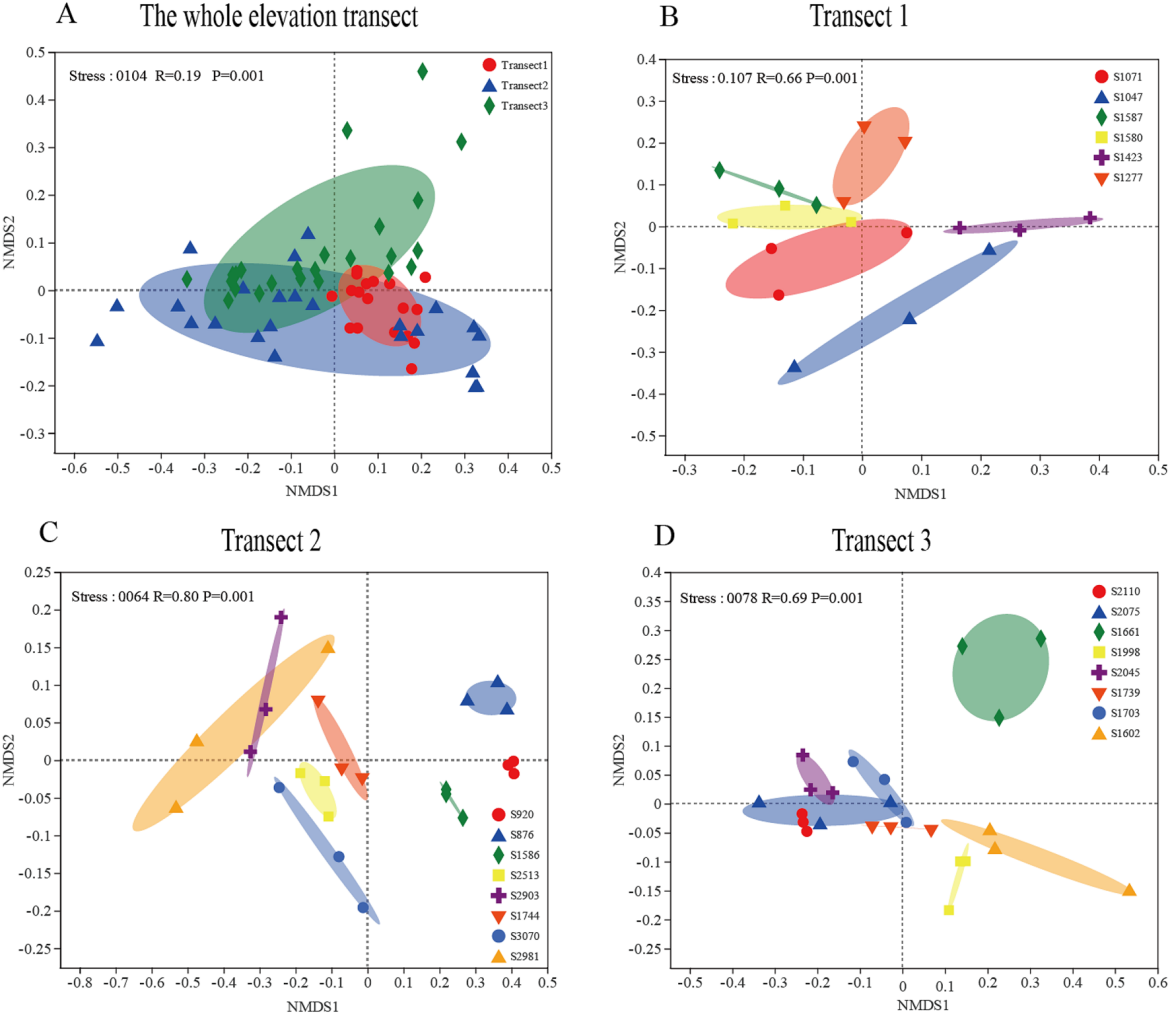
Nonmetric multidimensional scaling (NMDS) analysis of bacterial community composition as a function of elevation sites across all samples sites (**A**), elevational Transect 1 (**B**), elevational Transect 2 (**C**), and elevational Transect 3 (**D**). The NMDS analysis was performed on the Bray Curtis similarity matrix, calculated based upon total OTUs. To corroborate the NMDS results, a one-way ANOSIM (analysis of similarities) was used to test the relationship effects of elevation on bacterial community beta diversity. These were done in R with the vegan package (v3.3.1, http://www.R-project.org).

A nonmetric multidimensional scaling analysis (NMDS) was next conducted to evaluate overall bacterial community structure composition at the OTU level. As shown in Fig. 4A, two small-scale elevational samples (1047–1587 m and 1602–2110 m) were significantly separated from one another (ANOSIM test, stress = 0.0104, R = 0.19, *P* = 0.001), and the elliptical coverage of the broader-scale elevation samples (Transect 2, 876–3070 m) partially overlapped with that of these two small-scale elevation samples (Transect 1, 1047–1587 m and Transect 3, 1602–2110 m). Overall, there were significant changes in the bacterial community structure in the three analyzed transects (Fig. 4B–D), except for several neighboring sites in the 1602–2110 m elevation range (S1703, S1739, S2045, S2075, and S2110). This suggests that elevation had the most significant impact on bacterial community structure in the Mt. Tianshan grassland.

### Relationships between bacterial community structures and environmental variables

**Figure 5:**
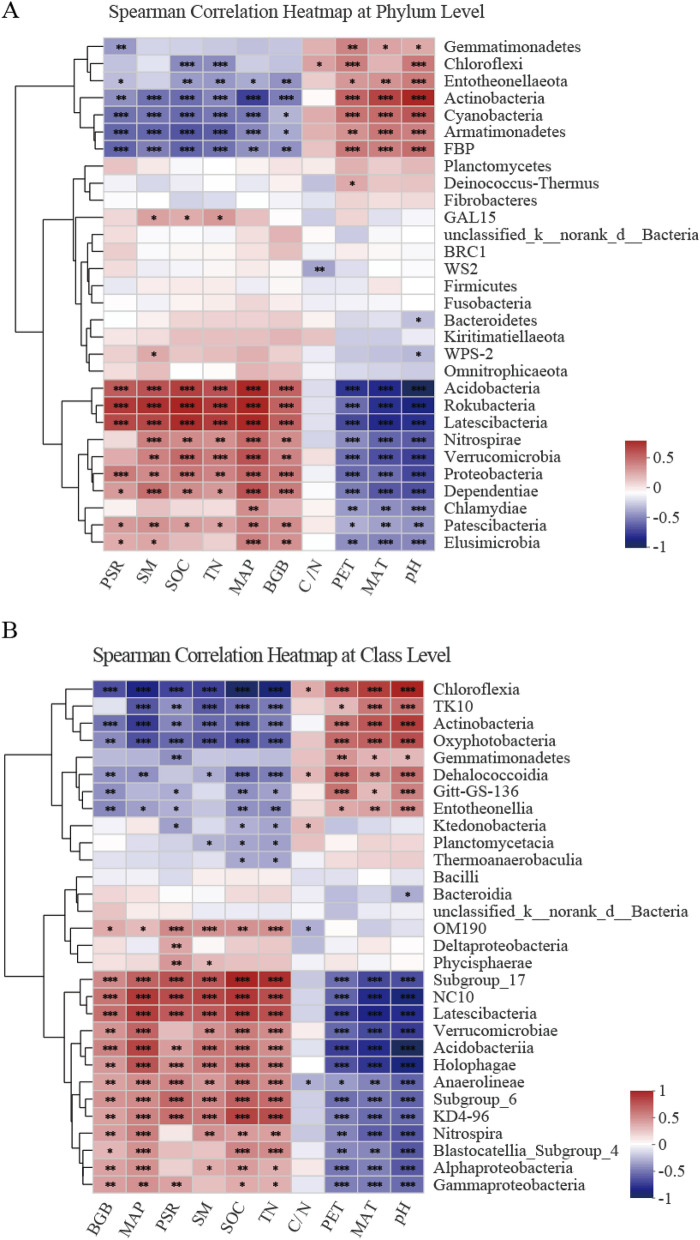
Spearman correlation analyses of the relationships between environmental variables and the top 30 bacterial phyla (**A**) or classes (**B**). Red and blue respectively denote positive and negative correlations. *P < 0.05, **P < 0.01, ***P < 0.001. These were done in R with the pheatmap package (v3.3.1, http://www.R-project.org).

Lastly, we evaluated correlations between environmental variables and the top 30 soil bacterial phyla, classes and genera using heatmaps, revealing that the soil bacterial community composition was simultaneously affected by climatic, plant, and soil physicochemical factors (Fig. 5; Supplementary Fig. 2). The relationships between the environmental variables and relative abundance varied as a function of bacterial phylum and class which are affected differently by environmental factors. As shown in Fig. 5, Spearman correlation analysis revealed that soil pH and mean annual temperature (MAT) were the key factors influencing on the relative abundances of the main bacterial phyla and classes. The abundance of *Actinomycetes*, *Gemmatimonadetes* and *Chloroflexi* phyla, as well as the diversity variables, were significantly and positively correlated with both soil pH and PET (Fig. 5A; Table 2; Supplementary Fig. 2; *P* < 0.001), while *Acidobacteria*, *Proteobacteria* and *Verrucomicrobia* showed significant negative correlations with soil pH and (potential evapotranspiration) PET. Additionally, at the genus level, MAT, PET, and pH were also significantly correlated with bacterial genera (Supplementary Fig. 2).

**Table 2:** Pearson correlations (R) evaluating the association between environmental variables and bacterial diversity (Chao1 richness, Shannon–Wiener diversity).

R	Elevation	pH^a^	MAT	MAP	PET	C/N	SM	SOC	TN	BGB	PSR
Chao1 index	**− 0.428**^b^**	0.418**	0.453**	− 0.380**	0.385**	− 0.222	− 0.226	− 0.118	− 0.019	− 0.270*	−0.157
Shannonindex	− 0.456**	0.381**	0.479**	− 0.404**	0.437**	− 0.179	− 0.301*	− 0.187	− 0.077	− 0.319**	−0.154

^a^MAP, MAT, PET, C/N, SM, SOC, TN, BGB, and PSR respectively correspond to mean annual precipitation, mean annual temperature, evapotranspiration, carbon/nitrogen ratio, soil moisture, total carbon, belowground biomass, and plant species richness.

^b^Bold values indicate significant correlations (*P* < 0.05).

## Discussion

### Elevation patterns of bacterial community

In this study, we detected a hump-shaped richness/diversity pattern along a broad-scale elevation gradient (from 876 to 3070 m) in the Mt. Tianshan grassland ecosystem, although consistent patterns were not observed along elevation gradients in the three analyzed transects. Other studies have also observed hollow-shaped bacterial community distribution patterns at higher elevations (1820–4050 m) on Laojun Mountain, China^15^, while a hump-shaped bacterial diversity pattern was observed at between elevations of 1000–3700 m on Mount Fuji in Japan^8^, with maximal diversity occurring at 2500 m. In the present study, we observed maximal bacterial richness/diversity at 1277 m, which may be attributable to differences in baseline environmental variables in these different ecosystems^12^. Other studies have reported no significant elevation-related trends^5,7^ or decreasing elevation patterns^12^. One study reported no apparent elevational pattern in bacterial diversity along a broad elevational gradient (530–2200 m) on Changbai Mountain, China, whereas, in a narrow gradient (2000–2500 m) above the 1950 m baseline, a monotonically decreasing elevation pattern was detected^16^. A U-shaped diversity elevation pattern was found under 1770 m, whereas above this altitude a decreasing diversity pattern was observed with increasing elevation^17^. These prior findings underscore the profound potential impact of study scales and sampling schemes on observed elevation patterns, showing the importance of using broader elevation scales when exploring bacterial community elevational patterns.

The choice of proper scale plays an important role in researching environmental variables and microbial community patterns. Narrow-scale elevational gradients provide a more precise description of the changes in bacterial community structures than broader scales. Nevertheless, changes in some phyla occur at specific altitudes, for example, in Transect 3 at S1739 and S1998 there was an increase in the relative abundance of *Actinobacteria* and a decrease in *Proteobacteria* and *Acidobacteria*. This was not detected in the broad scale Transect 2. In this environment, at the low altitudes of Transect 1, it is not necessary to include a six-point of altitude scale because the differences in phyla abundance are not significant. Conversely, bacterial diversity needs broad altitudinal scales to show a consistent variation pattern. The scale (number of sampled points at different elevations) depends on each specific mountain environment and the specific altitude.

The exact factors responsible for the observed variability in elevation diversity patterns in these earlier studies remain to be clarified. We hypothesize that the use of broad elevation scales, as well as ecosystem-specific differences, may have contributed to these differences. The generality of these observed patterns across different habitats remains to be examined in future studies.

### Environmental factors affecting bacterial community composition

**Figure 6:**
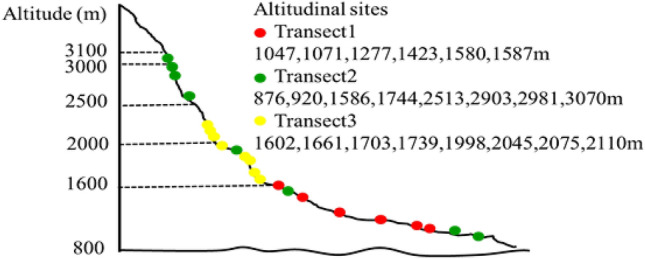
The locations of selected Mt. Tianshan grassland study sites along an elevation gradient. It was created using Adobe Illustrator CS6 (Adobe Software, USA).

In the present study, we observed a significant correlation between bacterial community composition and soil pH, with MAT being the factor next most closely related to bacterial diversity (Fig. 6). In addition to elevation, soil pH, MAT, and MAP were all significantly correlated with bacterial diversity (*P* < 0.05; Table 2). Soil pH has been reported to be the strongest predictor of bacterial diversity along elevational gradients^18^. Consistent with this, Shen et al.^9,12^ found that pH plays the largest role in modulating bacterial community variations on Changbai Mountain. We additionally found a secondary role for MAT and belowground root biomass (BGB) as regulators of such diversity when soil pH was not constant. Nottingham et al.^6^ found MAT to be the primary determinant of bacterial elevational diversity patterns in situations with constant soil pH. Temperature is often correlated with BGB and soil pH, indirectly affecting microbial communities, thus potentially explaining these results^19,20^.

Elevational scale is an important factor to consider when interpreting the results of this and similar studies. Elevation has been reported to be indicative of bacterial community diversity in soil and sediment environments^3,7,15,21^. Sampling a broad soil pH range across study sampling sites is a prerequisite for detecting pH-related differences in bacterial community compositions. If the soil pH range in this study had been narrower, MAT would likely have been the dominant determinant of relative bacterial abundance. Prior elevational studies on different mountains have reported soil pH ranges that were either wholly acidic or alkaline, whereas we found that the pH range in our study varied from acidic to alkaline (5.02–8.83), thus incorporating a broader range. Other environmental variables were also highly variable across study sites, likely reflecting the relatively complex conditions associated with these different elevations in general.

The analysis of bacterial composition revealed that *Actinobacteria*, *Proteobacteria* and *Acidobacteria* were the dominant phyla across the different transects and elevational sites on the Mt. Tianshan (Fig. 1), with significant differences in soil bacterial community composition between transects (Fig. 2). Changes in the bacterial community composition as a function of elevation have previously been thought to be attributable to habitat filtering as temperatures are lower at higher elevations^15^ and soil pH can vary across elevational study sites^16^.

In this study, we found considerable variation in the climate, plant, and soil properties which all varied dramatically across the sampled sites (Tables 1; Supplementary Table 1). The relative abundances of the phyla *Acidobacteria*, *Actinobacteria*, *Gemmatimonadetes*, and *Chloroflexi* were significantly correlated with pH (Fig. 5), consistent with results from prior analyses of montane biomes conducted using horizontal^10^ (at the continental scale across North and South America) and vertical^7^ (100–1950 m on Mt. Halla, South Korea) research scales. These results were in line with those of studies conducted on the Changbai Mountain, such as the article published by Shen et al.^9^, which found that the *Acidobacteria*, *Actinobacteria*, *Alphaproteobacteria*, and *Betaproteobacteria* abundance varied as a function of elevation, and that alterations in community composition were significantly correlated with soil pH, C/N, and moisture. In other studies, the *Acidobacteria* abundance has been shown to be significantly correlated with soil pH^22^, with these bacteria preferentially inhabiting neutral alkaline soil environments^23^. There are two likely explanations for why pH was the best predictor of bacterial community composition^10^. First, pH imposes direct physiological stress on bacterial growth^24^. Second, pH can also serve as an integrated functional index for other proximal factors that may influence local bacterial communities given that many different factors are directly or indirectly associated with soil pH^21,24^.

## Conclusion

In this study, the altitudinal patterns of bacterial composition and diversity observed varied significantly with elevation scale in the mountain grassland of Mt. Tianshan. The bacterial diversity exhibited a hump-shaped pattern when evaluating the overall elevation gradient, whereas no consistent pattern was detected on a narrower-scale. While bacterial community elevation patterns were significantly responsive to mean annual temperature (MAT) and potential evapotranspiration (PET) throughout the entire elevation gradient, they were more strongly affected by soil pH, which also influenced the elevational distribution pattern of soil bacterial diversity. The composition and diversity of soil bacteria exhibited niche differentiation characteristics in response to difference in soil pH, which is a primary consequence of heterogeneity in the soil environment. In conclusion, the changes in soil pH have a crucial role in shaping the composition and diversity of bacterial communities in the grassland ecosystem of Mt. Tianshan, China.

## Materials and methods

### Study region and site description

The chosen study site was situated west of Mt. Tianshan in northwest China, which is characterized by a typically arid continental temperate monsoon climate. In the present study, soil samples were collected from 22 representative sites. Each of the 22 sites was classified into three different transects (Fig. 6). There were six sites located in Transect 1 while both Transects 2 and 3 contained eight sites. Details of the sampled sites are shown in Supplementary Table 1.

### Sampling design and data collection

Soil samples were collected in July 2017. Briefly, a 100 m × 100 m sampling plot was established at each sampling site and the spatial geographic coordinates and elevation were recorded by using an eTrex Venture global positioning system (Garmin, USA). Three random 1 m × 1 m quadrats at each site were then selected, and soil samples (depth: 0–10 cm) were collected from these locations. In addition, the aboveground and belowground plant biomass from each quadrat were collected and sorted by species to compute plant species richness. After collection, samples were placed in sterile polyethylene bags, refrigerated in portable refrigeration units, and transported to the laboratory for further analysis. Soil samples were sieved through a 2.0 mm mesh to remove roots and other residue, and were then subdivided into two portions, one of which was maintained at 4 °C for measurements of soil properties, while the other was stored at − 80 °C before DNA extraction.

Data regarding mean annual temperature (MAT), mean annual precipitation (MAP), and potential evapotranspiration (PET) were obtained from the WorldClim database (https://www.worldclim.org). An IQ150 pH meter (Spectrum Technologies, USA) was used to measure soil pH at a 1:5 soil water ratio. A gravimetric approach was used to determine soil moisture after drying for 24 h in a 105 °C oven. Total nitrogen (TN) and total carbon (TC) were quantified with an Elemental Analyzer 3000 (Euro Vector, Italy). Soil inorganic carbon concentration was measured volumetrically using a Calcimeter 08.53 (Eijkelkamp, The Netherlands). The soil organic carbon levels were determined by calculating the difference between total carbon and soil inorganic carbon concentrations.

### DNA extraction and 16S sequencing

A DNeasy Power Soil Kit (QIAGEN, The Netherlands) was used to extract DNA from a 1.0 g aliquot of each soil sample according to provided directions, after which a NanoDrop ND- 1000 instrument (Thermo Fisher Scientific, USA) and agarose gel electrophoresis, were used to evaluate DNA quantity and quality, respectively.

Research has shown that the V3–V4 region of the bacterial 16S rRNA gene provides high coverage and taxonomic accuracy with lower overestimation of alpha diversity^25^. Therefore, the 338F (5′-ACTCCTACGGGAGGCAGCA-3′) and 806R (5′-GGACTACHVGGGTWTCTAAT-3′) primers^26^ were used to amplify the bacterial V3–V4 region, with sample-specific 7–bp barcode sequences incorporated into the primers to facilitate multiplexed sequencing. The thermocycler settings were as follows: 98 °C for 2 min, 25 cycles of 98 °C for 15 s, 55 °C for 30 s, and 72 °C for 30 s, followed by 72 °C for 5 min. Agencourt AMPure beads (Beckman Coulter, USA) were used to amplify the purified PCR products, which were quantified with a PicoGreen dsDNA Assay Kit (Invitrogen, USA). Equal amounts of all amplicons were then pooled, and paired-end 2 × 300 bp sequencing was conducted by Shanghai Personal Biotechnology (China) with an Illumina MiSeq PE250 instrument and the MiSeq Reagent Kit v3.

### Sequencing analysis

After removing the sequence adapters, the pair-end sequences were merged into full-length sequences using FLASH v1.2.3^27^. Then the quantitative insights into microbial ecology QIIME pipeline (v1.8.0) was employed to process the sequencing data as previously described^28^. After denoising and chimera removal using DADA2^29^, the remaining high-quality sequences were clustered into operational taxonomic units (OTUs) with a similarity greater than 97% using an UCLUST model^30^. Representative sequences for each OTU were selected by default parameters, and a BLAST search against the Greengenes database^31^ was used for the OTU classification of these representative sequences based upon the best match. OTU tables were generated to present OTU abundance in each sample and to detail OTU taxonomy. Those OTUs that contained fewer than 0.001% of overall sequences across all samples were discarded. To avoid bias caused by different sequencing depth, sequences were resampled to the sample sequencing depth (3167 sequences per sample) across all samples.

### Bioinformatics and statistical analyses

The QIIME v1.8.0 pipelines (http://qiime.sourceforge.net)^28^ and R v3.3.1 packages (R Foundation for Statistical Computing; available at http://www.R-project.org) were used for sequencing data analysis. OTU-level alpha indices of alpha diversity including the Chao1 index and the Shannon–Wiener index were calculated using the QIIME OTU table. Analysis of similarities (ANOSIM) was performed based on the Bray–Curtis dissimilarity by non-metric multidimensional scaling (NMDS) using the “vegan” package^32^. The pairwise Spearman’s correlation matrix between environmental factors and bacteria was performed by the “psych” package^33^. Heatmap plots were generated using the “pheatmap” package (R Development Core Team, https://CRAN.R-project.org/package=pheatmap)^34^.

Data are given as means ± standard error (SE) for three replicate samples. One-way analysis of variance and Duncan's multiple comparisons were used to determine the differences between treatments regarding soil physicochemical and microbial community abundance. Statistical analyses were performed using SPSS 20.0 (IBM, Corp, Armonk, NY, USA), and SigmaPlot 10.0 (Systat Software, Inc., San Jose California USA, www.systatsoftware.com).

## Supplementary Information

Supplementary Information.

## Data Availability

All data included in this study are available upon request by contact with the corresponding author.
