# Supplementary material for: Distinct soil bacterial patterns along narrow and broad elevational gradients in the grassland of Mt. Tianshan, China
Source: Sci Rep. 2022 Jan 7;12:136. doi: 10.1038/s41598-021-03937-x (PMC8742048; doi:10.1038/s41598-021-03937-x)
Supplement: Supplementary file 1 — Supplementary Information. [file 41598_2021_3937_MOESM1_ESM.doc]

**Distinct soil bacterial patterns along narrow and broad elevational gradients in the grassland** [**of Mt. Tianshan, China**](https://link.springer.com/article/10.1007/s40333-014-0070-0)

Supplementary Table 1 Summary of sampling site characteristics.

| Elevation  (m) | Latitude  (N) | Longitude  (E) | MAT  ( ^o^C) | MAP  (mm) | PET  (mm) | Soil types | Dominant plant species |
| --- | --- | --- | --- | --- | --- | --- | --- |
| Transect 1 |  |  |  |  |  |  |  |
| 1047 | 81°31′ | 44°07′ | 8.07 | 334.0 | 955 | Sierozem soil (SZS) | *Gramineae**, Leguminosae* |
| 1071 | 81°31′ | 44°10′ | 7.76 | 328.2 | 944 | Chestnut soil (CNS) | *Gramineae* |
| 1277 | 81°35′ | 44°13′ | 6.61 | 347.3 | 880 | Chestnut soil (CNS) | *Cannabis,* *Carex* sp*., Galium* sp. |
| 1423 | 81°37′ | 44°16′ | 6.55 | 335.0 | 835 | Chestnut soil (CNS) | *Gramineae* |
| 1580 | 81°38′ | 44°15′ | 6.38 | 338.6 | 842 | Chernozem soil (CZS) | *Phlomis pratensis*, *Leymus*, *Gramineae* |
| 1587 | 81°38′ | 44°15′ | 6.15 | 348.6 | 824 | Chernozem soil (CZS) | *Phlomis pratensis*, *Salvia japonica Thunb.* |
| Transect 2 |  |  |  |  |  |  |  |
| 876 | 81°07′ | 43°40′ | 8.05 | 269.8 | 1036 | Sierozem soil (SZS) | *Seriphidium transiliense*, *Ceratocarpus arenarius* |
| 920 | 81°09′ | 43°39′ | 7.96 | 273.5 | 1025 | Sierozem soil (SZS) | *Seriphidium transiliense* |
| 1586 | 81°07′ | 43°30′ | 6.12 | 351.7 | 889 | Chestnut soil (CNS) | *Stipa,  Gramineae,  Compositae* |
| 1744 | 81°07′ | 43°29′ | 5.46 | 372.3 | 850 | Chernozem soil (CZS) | *Phlomis pratensis,Iridaceae,* |
| 2513 | 81°04′ | 43°26′ | 2.67 | 457.4 | 713 | Mountain meadow soil (MMS) | *Carex* sp*., Phlomis pratensis,Alchemilla tianshanica Juz*. |
| 2903 | 81°03′ | 43°24′ | 1.02 | 507.7 | 588 | Alpine meadow soil (AMS) | *Kobresia* |
| 2981 | 81°01′ | 43°21′ | 1.30 | 509.2 | 608 | Alpine meadow soil (AMS) | *Carex* sp*.* |
| 3070 | 81°02′ | 43°23′ | 1.79 | 487.6 | 601 | Alpine meadow soil (AMS) | *Alchemilla tianshanica Juz*., *Carex* sp*.*, *Stipa* |
| Transect 3 |  |  |  |  |  |  |  |
| 1602 | 81°28′ | 43°09′ | 5.06 | 420.6 | 897 | Chestnut soil (CNS) | *Gramineae* |
| 1661 | 80°51′ | 42°51′ | 5.03 | 409.9 | 923 | Chestnut soil (CNS) | *Achnatherum* |
| 1703 | 81°23′ | 43°08′ | 4.46 | 450.9 | 890 | Chestnut soil (CNS) | *Stipa*, *Thalictrum simplex* |
| 1739 | 81°17′ | 43°03′ | 4.15 | 470.3 | 880 | Chestnut soil (CNS) | *Stipa* |
| 1998 | 80°35′ | 42°39′ | 4.70 | 383.0 | 866 | Chernozem soil (CZS) | *Phlomis pratensis*, *Stipa* |
| 2045 | 80°34′ | 42°39′ | 4.52 | 384.7 | 840 | Chernozem soil (CZS) | *Bromus*, *Phlomis pratensis* |
| 2075 | 81°7′ | 43°14′ | 2.75 | 508.7 | 789 | Chernozem soil (CZS) | *Gramineae* |
| 2110 | 81°5′ | 43°15′ | 2.75 | 506.9 | 771 | Chernozem soil (CZS) | *Geranium pratense*, *Pedicularis reaupinanta L.* |

MAP, mean annual precipitation. MAT, mean annual temperature. PET, potential evapotranspiration.

Supplementary Table 2 Pearson correlations (R) between the relative abundances of the bacterial phylum and environment factors.

| **Correlation R** | **MAP** | **MAT** | **PET** | **pH** | **C/N** | **SM** | **SOC** | **TN** | **BGB** | **PSR** |
| --- | --- | --- | --- | --- | --- | --- | --- | --- | --- | --- |
| **Actinobacteria** | -0.64 | 0.63 | 0.49 | **0.72** | -0.03 | -0.48 | -0.49 | -0.41 | -0.42 | -0.39 |
| **Proteobacteria** | 0.49 | -0.49 | -0.54 | **-0.61** | -0.14 | 0.34 | 0.41 | 0.37 | 0.46 | 0.42 |
| **Acidobacteria** | 0.72 | -0.71 | -0.65 | **-0.85** | -0.16 | 0.58 | 0.69 | 0.61 | 0.53 | 0.58 |
| **Chloroflexi** | -0.27 | 0.29 | 0.46 | **0.52** | 0.35 | -0.18 | -0.56 | -0.57 | -0.39 | -0.37 |
| **Gemmatimonadetes** | -0.22 | 0.24 | 0.41 | 0.25 | 0.20 | -0.15 | -0.19 | -0.20 | -0.24 | -0.35 |
| **Verrucomicrobia** | 0.57 | -0.59 | -0.48 | **-0.63** | 0.04 | 0.38 | 0.50 | 0.43 | 0.39 | 0.27 |
| **Bacteroidetes** | 0.15 | -0.22 | -0.29 | -0.28 | -0.03 | 0.08 | 0.24 | 0.23 | 0.29 | 0.11 |
| **Planctomycetes** | -0.05 | 0.16 | 0.34 | 0.30 | 0.03 | 0.00 | -0.11 | -0.08 | -0.08 | 0.10 |
| **Firmicutes** | -0.10 | 0.05 | -0.07 | -0.03 | -0.02 | -0.02 | -0.03 | -0.04 | -0.12 | -0.17 |
| **Rokubacteria** | 0.76 | -0.73 | -0.52 | **-0.76** | -0.11 | 0.70 | 0.76 | 0.68 | 0.50 | 0.65 |
| **Cyanobacteria** | -0.40 | 0.38 | 0.36 | **0.47** | 0.30 | -0.46 | -0.52 | -0.50 | -0.23 | -0.55 |
| **Nitrospirae** | 0.35 | -0.42 | -0.35 | **-0.43** | -0.22 | 0.32 | 0.28 | 0.30 | 0.23 | 0.08 |
| **Entotheonellaeota** | -0.20 | 0.24 | 0.28 | **0.35** | 0.12 | -0.11 | -0.24 | -0.25 | -0.28 | -0.14 |
| **unclassified** | 0.03 | -0.02 | -0.10 | -0.03 | -0.04 | -0.09 | -0.02 | -0.06 | 0.05 | 0.11 |
| **Latescibacteria** | 0.73 | -0.76 | -0.65 | **-0.77** | -0.22 | 0.60 | 0.76 | 0.72 | 0.49 | 0.62 |
| **Patescibacteria** | 0.35 | -0.32 | -0.37 | **-0.43** | -0.19 | 0.29 | 0.22 | 0.23 | 0.37 | 0.22 |
| **Armatimonadetes** | -0.29 | 0.25 | 0.19 | 0.28 | 0.14 | -0.42 | -0.48 | -0.47 | -0.27 | -0.45 |
| **Elusimicrobia** | 0.28 | -0.30 | -0.40 | -0.28 | -0.19 | 0.11 | 0.12 | 0.12 | 0.27 | 0.14 |
| **WPS-2** | 0.03 | -0.06 | -0.05 | -0.18 | -0.04 | -0.11 | -0.08 | -0.02 | 0.02 | 0.05 |
| **Chlamydiae** | 0.49 | -0.48 | -0.43 | **-0.57** | 0.03 | 0.41 | 0.48 | 0.39 | 0.59 | 0.43 |
| **Dependentiae** | 0.52 | -0.50 | -0.42 | **-0.54** | 0.00 | 0.38 | 0.37 | 0.29 | 0.50 | 0.36 |
| **WS2** | 0.11 | -0.19 | -0.16 | -0.22 | -0.19 | -0.03 | 0.27 | 0.31 | 0.15 | 0.14 |
| **FBP** | -0.37 | 0.36 | 0.27 | **0.36** | 0.07 | -0.43 | -0.44 | -0.39 | -0.29 | -0.44 |
| **Deinococcus-Thermus** | -0.29 | 0.31 | 0.26 | **0.37** | 0.04 | -0.27 | -0.45 | -0.41 | -0.12 | -0.31 |
| **Fibrobacteres** | -0.17 | 0.25 | 0.29 | **0.33** | 0.05 | -0.11 | -0.23 | -0.22 | -0.12 | -0.09 |
| **BRC1** | -0.09 | -0.01 | -0.17 | -0.03 | 0.07 | -0.12 | -0.01 | -0.03 | 0.08 | -0.16 |
| **Fusobacteria** | 0.09 | -0.08 | -0.09 | -0.05 | -0.10 | -0.09 | -0.01 | 0.01 | 0.01 | -0.04 |
| **Omnitrophicaeota** | 0.25 | -0.22 | -0.21 | -0.26 | 0.02 | 0.24 | 0.08 | 0.04 | 0.25 | 0.17 |
| **GAL15** | -0.07 | 0.05 | -0.13 | 0.04 | -0.15 | -0.11 | 0.17 | 0.21 | 0.19 | 0.25 |
| **FCPU426** | 0.09 | -0.15 | -0.15 | -0.09 | -0.19 | -0.08 | 0.08 | 0.13 | -0.05 | -0.04 |

Notes: Values in bold indicate significant correlations (*P* < 0.05). The same as follow.

Supplementary Table 3 Pearson correlations (R) between the relative abundances of the bacterial classes and environment factors.

| **Correlation R** | **MAP** | **MAT** | **PET** | **pH** | **C/N** | **SM** | **SOC** | **TN** | **BGB** | **PSR** |
| --- | --- | --- | --- | --- | --- | --- | --- | --- | --- | --- |
| Actinobacteria | -0.64 | 0.63 | 0.50 | **0.74** | -0.03 | -0.47 | -0.50 | -0.41 | -0.44 | -0.39 |
| Alphaproteobacteria | 0.40 | -0.40 | -0.44 | **-0.54** | 0.08 | 0.29 | 0.35 | 0.29 | 0.33 | 0.17 |
| Gammaproteobacteria | 0.39 | -0.42 | -0.40 | -0.47 | -0.08 | 0.23 | 0.30 | 0.28 | 0.35 | 0.33 |
| Subgroup_6 | 0.42 | -0.44 | -0.46 | **-0.50** | -0.16 | 0.45 | 0.59 | 0.55 | 0.38 | 0.56 |
| Deltaproteobacteria | -0.06 | 0.07 | -0.05 | 0.01 | -0.23 | 0.02 | 0.18 | 0.19 | 0.11 | 0.32 |
| Gemmatimonadetes | -0.24 | 0.27 | 0.38 | 0.25 | 0.22 | -0.18 | -0.19 | -0.22 | -0.23 | -0.35 |
| Blastocatellia_Subgroup_4 | 0.40 | -0.38 | -0.37 | **-0.54** | -0.06 | 0.21 | 0.44 | 0.42 | 0.26 | 0.23 |
| Verrucomicrobiae | 0.57 | -0.59 | -0.48 | **-0.64** | 0.06 | 0.39 | 0.48 | 0.42 | 0.38 | 0.24 |
| KD4-96 | 0.45 | -0.47 | -0.40 | -0.48 | -0.15 | 0.49 | 0.71 | 0.66 | 0.34 | 0.52 |
| Chloroflexia | -0.72 | 0.72 | 0.62 | **0.82** | 0.30 | -0.62 | -0.79 | -0.75 | -0.56 | -0.60 |
| Acidobacteriia | 0.69 | -0.69 | -0.63 | **-0.84** | -0.10 | 0.51 | 0.51 | 0.41 | 0.46 | 0.35 |
| Bacteroidia | 0.11 | -0.14 | -0.22 | -0.27 | -0.06 | -0.02 | 0.13 | 0.14 | 0.15 | -0.01 |
| Bacilli | -0.05 | 0.00 | -0.10 | -0.08 | -0.10 | 0.06 | 0.07 | 0.06 | -0.05 | -0.12 |
| Anaerolineae | 0.41 | -0.39 | -0.31 | **-0.50** | -0.25 | 0.36 | 0.48 | 0.42 | 0.34 | 0.44 |
| NC10 | 0.74 | -0.70 | -0.50 | **-0.74** | -0.14 | 0.69 | 0.75 | 0.66 | 0.51 | 0.66 |
| Gitt-GS-136 | -0.24 | 0.25 | 0.52 | 0.46 | 0.07 | -0.12 | -0.35 | -0.30 | -0.35 | -0.26 |
| Planctomycetacia | -0.11 | 0.15 | 0.03 | 0.14 | 0.22 | -0.24 | -0.29 | -0.30 | 0.01 | -0.15 |
| TK10 | -0.54 | 0.50 | 0.25 | **0.53** | 0.15 | -0.54 | -0.47 | -0.42 | -0.10 | -0.35 |
| Holophagae | 0.64 | -0.66 | -0.56 | **-0.74** | 0.00 | 0.48 | 0.52 | 0.45 | 0.37 | 0.40 |
| Subgroup_17 | 0.58 | -0.59 | -0.47 | **-0.57** | -0.18 | 0.63 | 0.83 | 0.78 | 0.43 | 0.64 |
| Oxyphotobacteria | -0.56 | 0.59 | 0.55 | **0.66** | 0.21 | -0.54 | -0.57 | -0.51 | -0.37 | -0.48 |
| OM190 | 0.25 | -0.17 | 0.01 | -0.10 | -0.25 | 0.40 | 0.39 | 0.40 | 0.30 | 0.43 |
| Dehalococcoidia | -0.35 | 0.39 | 0.56 | **0.53** | 0.26 | -0.26 | -0.45 | -0.41 | -0.33 | -0.18 |
| Nitrospira | 0.47 | -0.51 | -0.39 | **-0.55** | -0.18 | 0.39 | 0.35 | 0.34 | 0.34 | 0.08 |
| Entotheonellia | -0.30 | 0.33 | 0.31 | 0.41 | 0.13 | -0.19 | -0.35 | -0.36 | -0.37 | -0.25 |
| Thermoanaerobaculia | -0.12 | 0.16 | 0.12 | 0.18 | -0.05 | -0.10 | -0.27 | -0.28 | -0.10 | -0.14 |
| Ktedonobacteria | 0.08 | -0.12 | -0.19 | -0.07 | 0.25 | -0.12 | -0.25 | -0.29 | -0.04 | -0.30 |
| Phycisphaerae | 0.13 | -0.04 | 0.08 | 0.01 | -0.05 | 0.25 | 0.22 | 0.21 | 0.11 | 0.37 |
| Latescibacteria | 0.73 | -0.72 | -0.61 | **-0.69** | -0.14 | 0.61 | 0.71 | 0.66 | 0.53 | 0.61 |
| unclassified_k__norank_d__Bacteria | 0.06 | -0.07 | -0.21 | -0.05 | -0.03 | -0.07 | -0.06 | -0.08 | 0.21 | 0.07 |

Supplementary Table 4 Pearson correlations (R) between the relative abundances of the bacterial dominant (Top 30) OTUs and environment variables.

| **Correlation R** | Genus | Class | **MAP** | **MAT** | **PET** | **pH** | **C/N** | **SM** | **SOC** | **TN** | **BGB** | **PSR** |
| --- | --- | --- | --- | --- | --- | --- | --- | --- | --- | --- | --- | --- |
| **OTU4579** | Rubrobacter | Actinobacteria | -0.01 | 0.02 | 0.03 | 0.04 | -0.43 | 0.09 | 0.20 | 0.28 | -0.05 | 0.20 |
| **OTU1815** | norank_f__67-14 | Actinobacteria | -0.22 | 0.27 | 0.21 | 0.32 | -0.25 | -0.01 | 0.05 | 0.13 | -0.11 | 0.18 |
| **OTU2984** | Bradyrhizobium | Alphaproteobacteria | 0.69 | -0.70 | -0.67 | **-0.79** | -0.15 | 0.57 | 0.74 | 0.67 | 0.52 | 0.55 |
| **OTU2951** | unclassified_f__Rhizobiaceae | Alphaproteobacteria | 0.12 | -0.22 | -0.34 | -0.33 | 0.07 | -0.07 | 0.12 | 0.10 | 0.23 | -0.10 |
| **OTU3755** | Solirubrobacter | Actinobacteria | -0.55 | 0.56 | 0.48 | **0.67** | -0.09 | -0.38 | -0.37 | -0.26 | -0.42 | -0.22 |
| **OTU5255** | Blastococcus | Actinobacteria | -0.74 | 0.69 | 0.44 | **0.69** | 0.02 | -0.72 | -0.64 | -0.55 | -0.51 | -0.53 |
| **OTU4397** | Ellin6055 | Alphaproteobacteria | -0.41 | 0.39 | 0.14 | 0.32 | -0.01 | -0.42 | -0.23 | -0.16 | -0.18 | -0.30 |
| **OTU291** | norank_f__Xanthobacteraceae | Alphaproteobacteria | 0.52 | -0.50 | -0.52 | **-0.62** | -0.21 | 0.47 | 0.66 | 0.65 | 0.38 | 0.41 |
| **OTU2987** | norank_f__norank_o__norank_c__KD4-96 | KD4-96 | 0.54 | -0.54 | -0.50 | **-0.57** | -0.38 | 0.57 | 0.77 | 0.76 | 0.47 | 0.69 |
| **OTU590** | Asanoa | Actinobacteria | -0.38 | 0.41 | 0.37 | 0.43 | -0.29 | -0.14 | -0.06 | 0.04 | -0.15 | -0.07 |
| **OTU3590** | norank_f__Gemmatimonadaceae | Gemmatimonadetes | 0.05 | -0.05 | 0.01 | -0.10 | 0.04 | 0.05 | 0.21 | 0.17 | 0.00 | -0.09 |
| **OTU3499** | norank_f__norank_o__norank_c__Gitt-GS-136 | Gitt-GS-136 | -0.47 | 0.51 | 0.68 | **0.69** | 0.07 | -0.30 | -0.55 | -0.47 | -0.50 | -0.38 |
| **OTU3198** | Candidatus_Udaeobacter | Verrucomicrobiae | 0.74 | -0.76 | -0.67 | **-0.85** | -0.01 | 0.63 | 0.70 | 0.62 | 0.51 | 0.45 |
| **OTU1492** | Conexibacter | Actinobacteria | 0.58 | -0.61 | -0.63 | **-0.71** | -0.09 | 0.53 | 0.76 | 0.70 | 0.52 | 0.41 |
| **OTU4081** | Pseudonocardia | Actinobacteria | -0.50 | 0.51 | 0.47 | **0.61** | -0.16 | -0.34 | -0.35 | -0.27 | -0.36 | -0.23 |
| **OTU3297** | norank_f__norank_o__norank_c__KD4-96 | KD4-96 | 0.33 | -0.38 | -0.40 | -0.42 | -0.27 | 0.42 | 0.64 | 0.65 | 0.34 | 0.49 |
| **OTU1865** | RB41 | Blastocatellia_Subgroup_4 | 0.40 | -0.42 | -0.34 | -0.45 | -0.24 | 0.44 | 0.70 | 0.72 | 0.35 | 0.44 |
| **OTU2727** | Microlunatus | Actinobacteria | 0.32 | -0.36 | -0.30 | -0.35 | -0.37 | 0.37 | 0.63 | 0.68 | 0.24 | 0.51 |
| **OTU4496** | Bacillus | Bacilli | 0.21 | -0.20 | -0.21 | -0.31 | -0.15 | 0.30 | 0.32 | 0.29 | 0.07 | 0.11 |
| **OTU2982** | unclassified_f__Micrococcaceae | Actinobacteria | -0.40 | 0.25 | -0.10 | 0.14 | 0.01 | -0.44 | -0.19 | -0.17 | -0.09 | -0.24 |
| **OTU215** | Actinoplanes | Actinobacteria | -0.59 | 0.55 | 0.28 | 0.48 | -0.03 | -0.50 | -0.40 | -0.33 | -0.26 | -0.46 |
| **OTU3104** | norank_f__norank_o__norank_c__Subgroup_6 | Subgroup_6 | 0.64 | -0.67 | -0.71 | **-0.80** | -0.08 | 0.55 | 0.69 | 0.61 | 0.53 | 0.49 |
| **OTU2938** | norank_f__Xanthobacteraceae | Alphaproteobacteria | 0.49 | -0.47 | -0.52 | **-0.63** | -0.32 | 0.47 | 0.71 | 0.69 | 0.44 | 0.63 |
| **OTU5076** | Solirubrobacter | Actinobacteria | -0.19 | 0.22 | 0.14 | 0.20 | -0.22 | -0.03 | 0.11 | 0.16 | -0.02 | 0.09 |
| **OTU4078** | unclassified_f__Ilumatobacteraceae | Actinobacteria | -0.25 | 0.23 | 0.34 | 0.41 | -0.12 | -0.01 | -0.13 | -0.06 | -0.26 | -0.09 |
| **OTU4749** | Rubrobacter | Actinobacteria | -0.58 | 0.60 | 0.52 | **0.66** | 0.06 | -0.52 | -0.68 | -0.63 | -0.50 | -0.46 |
| **OTU607** | Streptomyces | Actinobacteria | -0.76 | 0.77 | 0.51 | **0.66** | 0.02 | -0.51 | -0.49 | -0.42 | -0.28 | -0.42 |
| **OTU232** | Pseudonocardia | Actinobacteria | 0.39 | -0.44 | -0.52 | -0.44 | -0.32 | 0.36 | 0.48 | 0.46 | 0.29 | 0.37 |
| **OTU1754** | Solirubrobacter | Actinobacteria | 0.15 | -0.22 | -0.42 | -0.19 | -0.18 | 0.16 | 0.30 | 0.26 | 0.16 | 0.06 |
| **OTU5921** | Rhodoplanes | Alphaproteobacteria | 0.30 | -0.32 | -0.50 | -0.48 | -0.34 | 0.30 | 0.54 | 0.56 | 0.31 | 0.40 |

**
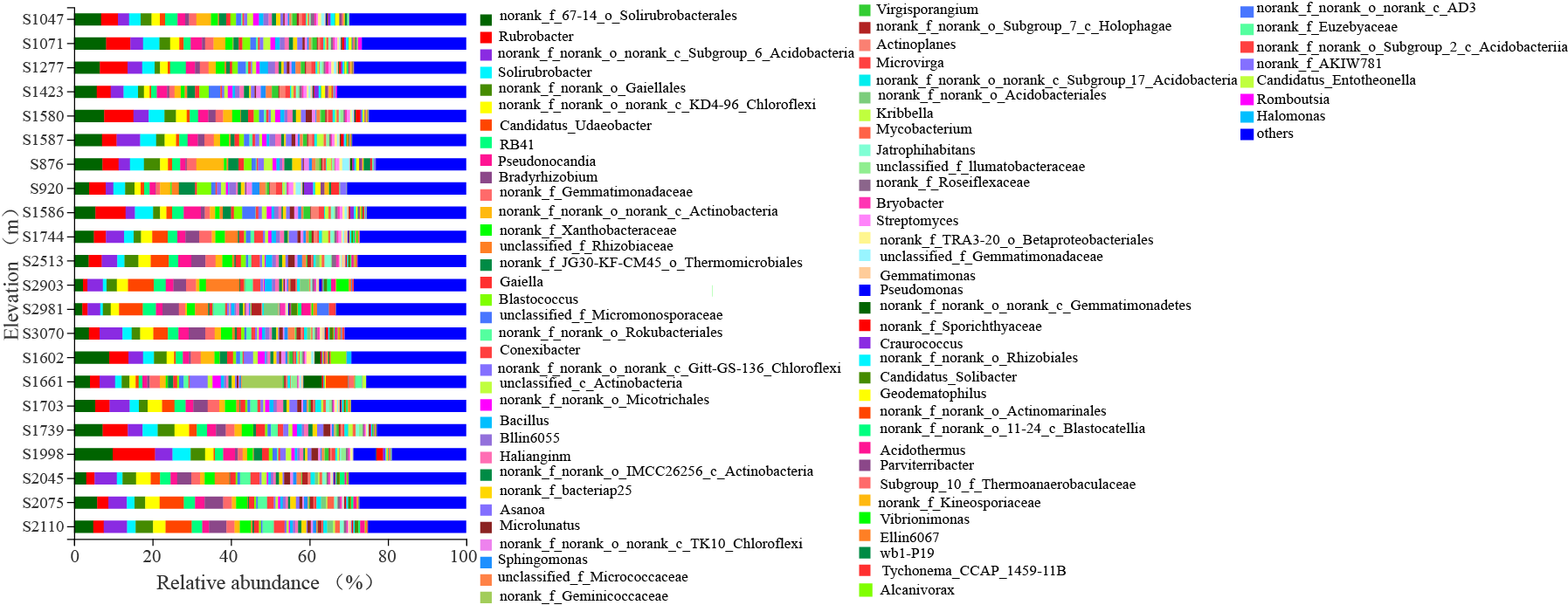
**

Supplementary Figure 1 The relative abundance of the detected genera of bacteria in samples from different elevation sites. Note: the Figure 1 and Figure 1 legend created using R v3.3.1 software (http://www.R-project.org).

**
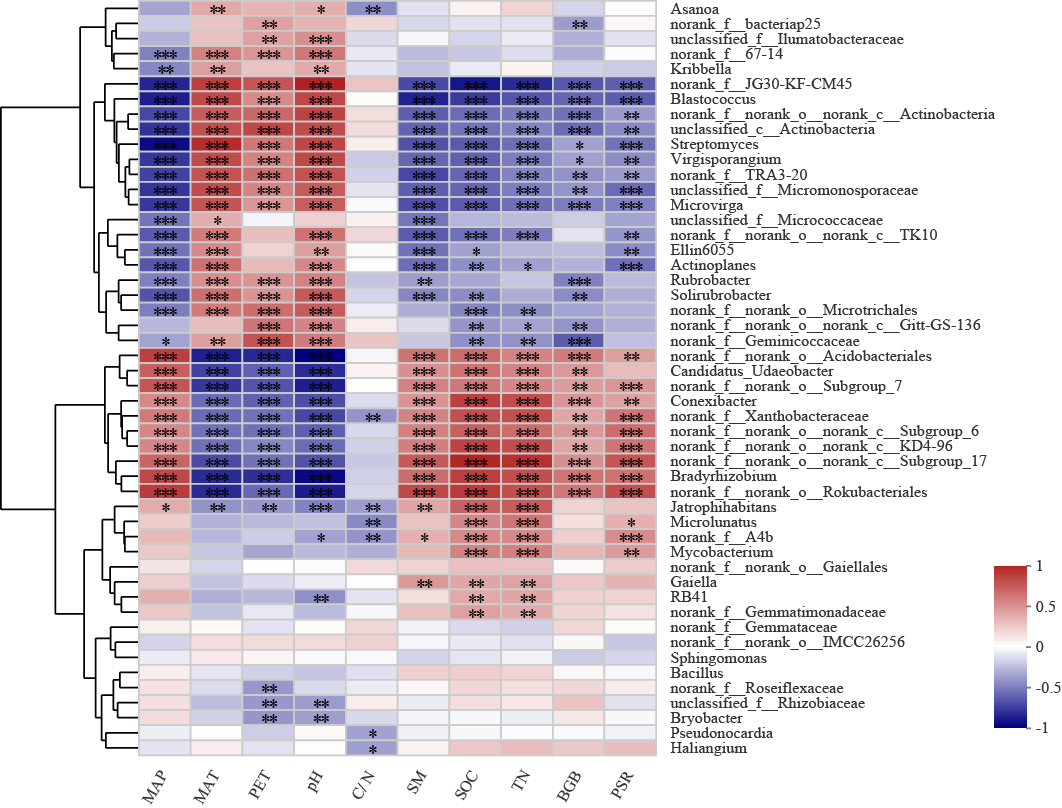
**

Supplementary Figure 2 Spearman correlation analyses of the relationships between environmental variables and bacterial genera. Note: Red and blue respectively denote positive and negative correlations. *P < 0.05, **P < 0.01, ***P < 0.001. Figure 2 and Figure 2 legend created using R v3.3.1 software (http://www.R-project.org).
